# Effects of post-exercise whey protein vs. whey protein plus creatine consumption in females

**DOI:** 10.1186/1550-2783-10-S1-P20

**Published:** 2013-12-06

**Authors:** Jordan Outlaw, Bailey Burks, Sara Hayward, Joshua Holt, Matt Stone, Brittany Stai, Brooke Cox, Cliffa Foster, Lem Taylor, Colin Wilborn

**Affiliations:** 1 Human Performance Laboratory, University of Mary Hardin-Baylor, Belton, TX 76513, USA

## Background

Creatine monohydrate is known to prolong time to fatigue and training volume during resistance training while ingestion of whey protein in the post-exercise window is critical to maximize adaptations. Individually, research supports that both creatine and whey protein ingestion ultimately leads to increased strength gains and improved body composition. Research is well supported and abundant in males, but research in a female population is limited overall and is much more limited when examining the effects of combined ingestion of whey protein plus creatine during resistance training. The purpose of this study was to examine the effects of an 8-week creatine plus whey protein supplementation and resistance training period on body composition and performance measures in young resistance-trained females.

## Methods

Eighteen (21 ± 2.5 yrs, 165.82 ± 6.45cm, 64.7 ± 8.2kg, 26.6 ± 4.78 % Body Fat) resistance-trained females were randomly assigned by lean body mass to Group A or B, ingesting whey protein (24g) or whey protein (24g) plus creatine monohydrate (5g), respectively, post-exercise in a single-blind manner. Subjects participated in a 4-day per week split body resistance training program for eight weeks. At baseline, 4 weeks, and 8 weeks, body composition (% body fat, lean mass, fat mass) measured by DEXA, muscular strength (leg press and bench press 1RM), muscular endurance, Wingate anaerobic power measurement (mean power, peak power), vertical, and broad jump measures were determined. Statistical analyses utilized a two-way ANOVA (group x time) with repeated measures for all dependent variables (*p* < 0.05).

## Results

A significant main effect for time was observed for % body fat (*p* = 0.007; Group A: -1.1556 ± 0.105%; Group B: -2.175 ± 0.171%) and lean mass (*p* = 0.000; A: 2532.445 ± 222.480g; B: 2520.85 ± 654.7g). No differences between groups were observed. No significant main effects for time or group were observed for changes in fat mass (*p* > 0.05). The performance variables broad jump (*p* = 0.001; A: 13 ± 2.529cm; B: 17.5 ± 6.139cm), vertical jump (*p* = 0.001; A; 0.112 ± 0.219in; B: 0.687 ± 0.257in), bench press (*p* = 0.000; A: 13.24 ± 1.514lb; B: 15.62 ± 1.991lb), leg press (*p* = 0.000; A: 217.23 ± 60.519lb; B: 176.25 ± 86.2lb), and Wingate mean power (*p* = 0.015; A: 39.37 ± 7.167W; B: 20.37 ± 10.351W) statistically increased over time but with no observed differences between groups. No significant main effects for time or group were observed for changes in Wingate peak power (*p* > 0.05).

## Conclusion

The primary findings of this study indicate adding creatine to post-workout protein ingestion does not enhance adaptations to an 8-week resistance training program in young resistance-trained females. Muscular strength, anaerobic power, and lean muscle mass all significantly increased after the 8-week training and supplementation protocol although there were no statistical differences between the two groups. This evidence suggests that resistance trained females may not receive an added benefit to creatine supplementation if protein supplementation is also occurring post-exercise.

